# Design, Immunogenicity and Preclinical Efficacy of the ChAdOx1.COVconsv12 Pan-Sarbecovirus T-Cell Vaccine

**DOI:** 10.3390/vaccines12090965

**Published:** 2024-08-26

**Authors:** Edmund G.-T. Wee, Sarah Kempster, Deborah Ferguson, Joanna Hall, Claire Ham, Susan Morris, Alison Crook, Sarah C. Gilbert, Bette Korber, Neil Almond, Tomáš Hanke

**Affiliations:** 1 The Jenner Institute, Nuffield Department of Medicine, University of Oxford, Oxford OX3 7DQ, UK; edmund.wee@ndm.ox.ac.uk (E.G.-T.W.); alison.crook@ndm.ox.ac.uk (A.C.); 2 Science and Research—Diagnostics, Medicines and Healthcare products Regulatory Agency, Potters Bar EN6 3QG, UK; sarah.kempster@mhra.gov.uk (S.K.); deborah.ferguson@mhra.gov.uk (D.F.); joanna.hall@mhra.gov.uk (J.H.); claire.ham@mhra.gov.uk (C.H.); neil.almond@mhra.gov.uk (N.A.); 3 Pandemic Sciences Institute, Nuffield Department of Medicine, University of Oxford, Oxford OX3 7DQ, UKsarah.gilbert@ndm.ox.ac.uk (S.C.G.); 4 New Mexico Consortium, Los Alamos, NM 87544, USA; bettekorber@newmexicoconsortium.org; 5 Joint Research Center for Human Retrovirus Infection, Kumamoto University, Kumamoto 860-0811, Japan

**Keywords:** COVID-19 vaccine, T cells, T cell vaccine, COVID-19 T cells, ChAdOx1, conserved region, Syrian hamster, challenge

## Abstract

During the COVID-19 pandemic, antibody-based vaccines targeting the SARS-CoV-2 spike glycoprotein were the focus for development because neutralizing antibodies were associated with protection against the SARS-CoV-2 infection pre-clinically and in humans. While deploying these spike-based vaccines saved millions of lives worldwide, it has become clear that the immunological mechanisms of protection against severe disease are multifaceted and involve non-neutralizing antibody components. Here, we describe a novel pan-sarbecovirus T-cell vaccine, ChAdOx1.COVconsv12, designed to complement and broaden the protection of spike vaccines. The vaccine immunogen COVconsv12 employs the two regions in the viral proteome most conserved among sarbecoviruses, which are delivered by replication-deficient vector ChAdOx1. It directs T cells towards epitopes shared among sarbecoviruses including evolving SARS-CoV-2 variants. Here, we show that ChAdOx1.COVconsv12 induced broad T-cell responses in the BALB/c and C57BL/6 mice. In the Syrian hamster challenge model, ChAdOx1.COVconsv12 alone did not protect against the SARS-CoV-2 infection, but when co-administered with 1/50th of the ChAdOx1 nCoV-19 spike vaccine protective dose, faster recovery and lower oral swab viral load were observed. Induction of CD8^+^ T cells may decrease COVID-19 severity and extend the T-cell response coverage of variants to match the known (and as yet unknown) members of the β-coronavirus family.

## 1. Introduction

Vaccine preparedness for future virus outbreaks is an essential element of public health protection highlighted by the pandemic of coronavirus disease 2019 (COVID-19). During the COVID-19 pandemic, the European Medicines Agency authorized 17 vaccines licensed against severe acute respiratory syndrome coronavirus 2 (SARS-CoV-2) and subsequently adapted two vaccines to match variants of concern better. These and nearly two hundred other vaccine candidates in clinical development have predominantly relied on the virus spike. This is because protection by licensed vaccines primarily focused on measuring the anti-spike neutralizing antibodies, which were associated with curbing the SARS-CoV-2 infection pre-clinically [1,2,3] and in human trials [4,5,6,7]. However, protection induced by the licensed SARS-CoV-2 vaccines and mediated by the spike-specific antibodies was typically narrow with limited coverage of other members of the same family, order, or genus, and their clinical effectiveness waned over time with the emergence of escape SARS-CoV-2 variants [8,9]. Other immunological mechanisms have been implied for protection against severe COVID-19 [10].

Multiple examples of protective CD8^+^ T cells from animal models and growing correlative data from vaccine usage suggest a critical contribution of cellular responses to the COVID-19 vaccines’ clinical efficacy. Thus, murine CD8^+^ T cells cross-protected against variants of concern [11,12]. In mice and monkeys challenged with SARS-CoV-2, CD8^+^ T cells provided disease protection, and their depletion led to increased virus replication [2,13]. In humans, the frequency of spike-specific CD8^+^ T cells but not neutralizing antibody titers positively correlated with the viral clearance rate [14]. CD4^+^ and CD8^+^ cytotoxic T cells displayed cross-reactivity against SARS-CoV-2 variants in convalescent donors [15,16], which were suggested to account for superior protection of mRNA-vaccinated individuals with prior SARS-CoV-2 infection, having so-called hybrid immunity, compared with mRNA vaccine alone [17]. A strong association between HLA-B*15:01 and asymptomatic SARS-CoV-2 infection was found, likely mediated by a pre-pandemic cross-reactive CD8^+^ T-cell memory response to seasonal coronaviruses [18]. In addition, old age was a strong predictor of COVID-19 severity [19], pointing to the well-demonstrated decline in naïve T cells in senescent individuals [20,21].

For groups of viruses with a significant epidemic and pandemic risk of spill-over zoonosis and subsequent human transmission, it is prudent to develop a vaccine that is potentially effective not only against the currently prioritized outbreak species but that can protect against all members of the entire taxonomic group. Unlike neutralizing antibodies, the induction of T cells offers the possibility of developing a vaccine that induces broad cross-reactive protection. The strategy is to focus vaccine-elicited T cells onto viral proteins’ shared, functionally conserved sub-protein regions [22,23,24]. In natural infection, immunodominant CD8^+^ T-cell responses are found against spike, matrix, and nucleocapsid [25]. However, these proteins are more variable [26] and, therefore, responses against them are ultimately less protective. Dagotto et al. first described the design of a T-cell vaccine immunogen employing the most highly conserved region of the sarbecovirus replication transcription complex, which they called conserved region 1 [11,27]. When delivered by a vector derived from rhesus adenovirus serotype 52, the RhAd52.CoV.Consv region 1 vaccination lowered viral loads in the nasal turbinates following a mouse-adapted SARS-CoV-2 challenge [11]. The present work builds on and endorses this approach using the CoV.Consv immunogen extended by additional conserved region 2 assembled into immunogen designated COVconsv12. We demonstrate the COVconsv12 preclinical immunogenicity in two strains of mice and assess its contribution to efficacy in Syrian hamsters when delivered by simian adenovirus-derived ChAdOx1, a vaccine vector with manufacturing capability sufficient to support the production of over three billion human doses during the COVID-19 pandemic.

## 2. Materials and Methods

### 2.1. Bioinformatic Analysis

We assembled 205 sarbecoviruses (Figure S1) representing much of the known virus diversity in wild animal populations from GenBank and GISAID [28,29]. A sarbecovirus full proteome alignment was generated starting with a MAAFT alignment [30], which was subsequently codon aligned, translated, and optimized in regions of insertions using AliView [31]. Only open reading frames were retained, only sequences that spanned the full proteome were included, and only small sequence sets representative of the diversity among highly sampled SARS-CoV-2 and SARS-CoV-1 sequences were utilized to avoid overrepresentation of these relatively highly conserved clades that entered and expanded within the human population. The maximum-likelihood phylogenetic tree was generated using the IQ tree [32] (Figure S2).

### 2.2. Cell Lines

M9 cells [33], a derivative from HEK 293T cells, were grown in DMEM10 (Dulbecco’s Modified Eagle Medium supplemented with L-glutamine and 10% fetal bovine serum (FBS) (GIBCO; #16250)). HeLa cells (ATCC number CCL-2; Manassas, VA, USA) were maintained in DMEM5. Cells were grown in a humidified incubator at 37 °C with 5% CO_2._

### 2.3. Vaccine Preparation

The COVconsv12 open reading frame based on the protein sequence of the SARS-CoV-2 Wuhan reference strain (Accession NC_045512) was synthesized (ThermoFisher Scientific, Paisley, UK) and inserted into the E1 region of the ChAdOx1 genome in the Bacterial Artificial Chromosome [23]. Linear ChAdOx1.COVconsv12 genomic DNA was excised using the *Pme*I restriction endonuclease and transfected into the adherent M9 cells [33] using Lipofectamine^TM^ CD2000 Transfection Reagent (ThermoFisher Scientific, Paisley, UK) following the vendor’s instructions [23]. The ChAdOx1.COVconsv12 virus was harvested, plaque-purified, and expanded for identity confirmation. Working virus stock was then grown in twelve T25 flasks, purified, titred, aliquoted, and stored at −80 °C until use as described previously [33].

The ChAdOx1 nCoV-19 and ChAdOx1.GFP vaccine stocks were prepared similarly [34].

### 2.4. Infection of HeLa Cells and Western Blot

HeLa cells were infected with ChAdOx1.COVconsv12 at indicated multiplicities of infection (MOI) and incubated at 37 °C, 5% CO_2_ for 1 day. Cells were collected, washed, lysed in the LDS Sample Buffer with NuPAGE Sample Reducing Agent (Invitrogen/ThermoFisher Scientific, Paisley, UK), incubated at 95 °C for 5 min, and placed on ice. The samples were then resolved with the NuPAGE electrophoresis system using the NuPAGE 4–12% Bis-Tris Gel (Invitrogen) and either stained with Coomassie brilliant blue or transferred onto Hamersham Hybond-LFP membranes (GE HealthCare Technologies Inc., Chicago, IL, USA) using the iBind Western Blot system (Invitrogen). The membranes were blocked with iBind solution with iBind addictive for 1 h, washed with phosphate-buffered saline (PBS) plus 0.1% Tween-20, and incubated with mouse monoclonal antibody (mAb) specific for the COVconsv12 C-terminal Pk-tag (generously provided by Prof. Richard E. Randall, St. Andrews University) for 1 h [35]. Bound primary antibodies were detected using alkaline phosphatase-conjugated anti-mouse mAb and visualized using the SigmaFast BCIP/NBT substrate (5-bromo-4-chloro-3-indolyl phosphate/nitro blue tetrazolium; Sigma-Aldrich Merck, Pool, UK).

### 2.5. Immunofluorescence

HeLa cells were infected with ChAdOx1.COVconsv12 at MOI 10 and incubated at 37 °C, 5% CO_2_ for 1 day. The cells were washed with PBS, fixed with 60% methanol/40% acetone, and blocked with 3% bovine serum albumin (BSA) in PBS for 30 min. Mouse anti-Pk tag mAb was then added for 1 h followed by goat anti-mouse FITC-conjugated mAb (Abcam, Cambridge, UK) in PBS plus 3% BSA for 1 h. After three washes with PBS, the coverslips were mounted on microscope slides using Vectashield DAPI nuclear stain mounting medium (Vector Laboratories, Newark, CA, USA). The slides were examined using a fluorescence microscope (Zeiss LSM 980 confocal microscope), and the images were analyzed using the Zeiss Zen 3.0 image analysis software.

### 2.6. Mice, Vaccinations and Preparation of Splenocytes and Lung Immune Cells

Six-week-old female BALB/c or C57BL/6 mice (Charles River, Harlow, UK) were immunized intramuscularly with 10^8^ virus particles (vp) of either the ChAdOx1.COVconsv12 or ChAdOx1 nCoV-19 vaccine alone or together at 10^8^ vp each, and the T-cell responses were analyzed 9 days later. At necropsy, splenocytes were isolated as described previously [36] and resuspended in R10 (RPMI 1640 medium supplemented with L-glutamine, 10% FCS, 1% penicillin/streptomycin and 50 µM β-mercaptoethanol) for the ELISPOT assay. To obtain immune cells from the lungs, the lungs were dissected, cut into 1-mm^3^ cubes, and digested with 1.4 mg/mL Collagenase Type XI from *Clostridium histolyticum* (Cat. No. C7657, Sigma-Aldrich, Gillingham, UK) and 60 µg/mL DNase Type IV (Sigma Aldrich) in 1.8 mL of R0 at 37 °C with shaking for 60 min. The reaction was stopped by adding 200 µL of FBS, and the cells were washed and resuspended in R10 for the ELISPOT assay.

### 2.7. Peptides

COVconsv12 peptides were 15-amino acid-long overlapping by 11 amino acids spanning the entire immunogen and were >90% pure by mass spectrometry (Synpeptide, Shanghai, China). All peptides were dissolved in DMSO (Sigma-Aldrich, Pool, UK) at 40 mg/mL, diluted to working stock solutions of 10 mg/mL, and stored at −80 °C until use.

### 2.8. Mouse IFN-γ ELISPOT Assay

As described before, a standard ELISPOT assay was performed using the mouse IFN-γ ELISpot kit (Mabtech, Stockholm, Sweden), with immune splenocytes tested separately from individual mice [37]. Briefly, ninety-six-well plates (Millipore, Watford, UK) had been pre-coated with 5-µg/mL anti-IFN-γ mAb AN18 (Mabtech, Stockholm, Sweden). One-hundred thousand splenocytes were added into each assay well and stimulated with peptides at the final concentrations of 2 μg/mL each at 37 °C, 5% CO_2_ for 18 h. Then, the cells were washed away with PBS, and the membranes were incubated with 1 µg/mL of biotinylated anti-IFN-γ mAb (Mabtech, Stockholm, Sweden) at room temperature for 2 h and 1 µg/mL of streptavidin-conjugated alkaline phosphatase (Mabtech, Stockholm, Sweden) at room temperature for 1 h with washing between. Spot-forming units (SFU) were visualized using a 10-min reaction with 5-bromo-4-chloro-3-idolyl phosphate and nitroblue tetrazolium (Bio-Rad, Richmond, CA, USA) and counted using the AID ELISpot Reader System (Autoimmun Diagnostika, Straßberg, Germany).

### 2.9. Hamster Vaccination and Challenge

Five groups of male and female Golden hamsters (five animals per group) were randomly allocated to treatment groups and administered intramuscularly once with the experimental ChAOx1-vectored vaccines (Table 1). (Note that the standard human dose of ChAdOx1 nCoV-19 was 5 × 10^10^ vp [34].) Four weeks later, the hamsters were challenged intranasally with 30 µL containing 1.5 × 10^5^ international units (IU) calibrated against the WHO RNA standard for SARS-CoV-2 RNA (NIBSC: 20/146) [38]. Victoria-1 equally distributed between the two nostrils. Victoria-1 (BetaCoV/Australia/VIC1/2020, Genbank: MT007544) has no coding mutations relative to Wuhan-Hu-1 (NC_045512). The original virus (passage 3) was received from Dr Mike Catton, Victorian Infectious Diseases Reference Laboratory, Melbourne, who isolated the virus from the first patient diagnosed with COVID-19 in Australia [39]. The virus was supplied by the Centre for AIDS Reagents (CFAR #100980), NIBSC, as with passage 4 grown in the VeroE6/TMPRSS2 cell line (CFAR #100978). All animals were weighed daily from the challenge to the end of the experiment. Two randomly selected hamsters from each group, one of each sex, were killed humanely at 6 days, a further two at 7 days, and the final animal from each group was humanely killed at 8 days post-challenge. The day of humane killing for each animal was allocated randomly prior to the start of the study.

### 2.10. RT-qPCR

Virus load was estimated by RT-qPCR. Oral swabs were collected from each hamster on days −2, 1, 2, 3, 4, and at necropsy (days 6, 7 or 8), and placed into Virus Transport Medium (VTM) (Hanks balanced salt solution with 2% heat-inactivated FBS, L-glutamine, 1% penicillin/streptomycin, and 0.5 µg/mL amphotericin B) for total nucleic acid extraction from a 200-µL sample using the MagnaPure24 (Roche, Basel, Switzerland) External Lysis Pathogen 200 protocol. Samples were eluted in a 50-µL volume.

At necropsy, tissue samples were collected from nasal turbinates, salivary glands, olfactory bulbs, and trachea, placed into RNA Later (ThermoFisher Scientific, Paisley, UK), and stored at −80 °C. On the analysis day, 20–50 mg of tissue was homogenized in 300 µL of R2 medium and the cell debris was removed by filtration. Total RNA was extracted using magnetic beads (MagnaPure 24, Roche, Basel, Switzerland) and adjusted to 20 ng/µL.

For both oral swabs and tissue, RT-qPCR was performed in triplicate using 5 µL of extracts per reaction and primers and probes targeting the envelope protein as described previously [40]. Viral shedding was expressed in IU per mL.

### 2.11. Hamster Pathology

Standard histological processes were used [41]. At post-mortem, intact left and right cranial lobes of lungs were collected and fixed in 10% neutral buffered formalin (Sigma-Aldrich, Pool, UK) at room temperature for 72 h and embedded in paraffin wax. Haematoxylin and eosin (H&E) staining and immunohistochemistry used 4-µm sections mounted on slides coated with poly-L-lysine. The tissue was de-waxed using xylene (ThermoFisher Scientific), stained with H&E, gradually re-hydrated using ethanol:water solutions (ThermoFisher Scientific), and analyzed for pathological changes associated with virus infection. Scores from 0 to 4 (0 = absent, 1 = minimal, 2 = mild, 3 = moderate, 4 = marked) for each variable were assigned independently by two veterinary pathologists blinded to the vaccination groups.

### 2.12. Hamster Immunohistochemistry

The Leica Bond Polymer Refine staining system (Leica Microsystems DS9800, Wetzlar, Germany) was used for the immunohistochemical staining. After the sliced sample de-waxing, IHC Protocol F was used with additional non-specific blocking before adding the primary antibodies (PBS supplemented with 10% normal horse serum (Biorad, Hercules, CA, USA), Casein (Vector Laboratories, Newark, CA, USA)) and extended 10-min haematoxylin staining time. Antibodies were diluted in the Bond primary diluent (Leica, Wetzlar, Germany; AR9352) and applied 1:1000 for the anti-SARS-CoV-2 spike protein mAb (Leica, AR9961) followed by rabbit polyclonal Ab 40150-T62-COV2-SIB (Sino Biologicals, Beijing, China), and 1:2000 for the anti-SARS-CoV-2 nucleoprotein mAb (Leica, AR9961) followed by mouse mAb 40143-MM05-SIB (Stratech Scientific/SinoBiologicals). Both primary antibodies were incubated for 30 min and the antigen unmasking was carried out in Heat-Induced Epitope Retrieval solution 1 (Leica AR9961) at 100 °C for 30 min.

### 2.13. Statistical Analysis

Statistical analyses were performed using Graph Pad Prism version 9.0 and SigmaPlot v12.5. For ELISPOT data, non-parametric tests were used, and the median (IQR) is shown. To facilitate the analysis of log-transformed data, samples with undetectable SARS-CoV-2 by RT-qPCR were assigned an arbitrary value of 20, representing the detection limit of the assay. Two-tailed *p* values were used, and *p* values of less than 0.05 were considered statistically significant.

## 3. Results

### 3.1. The ChAdOx1.COVconsv12 Vaccine Design and Construction

**Figure 1:**
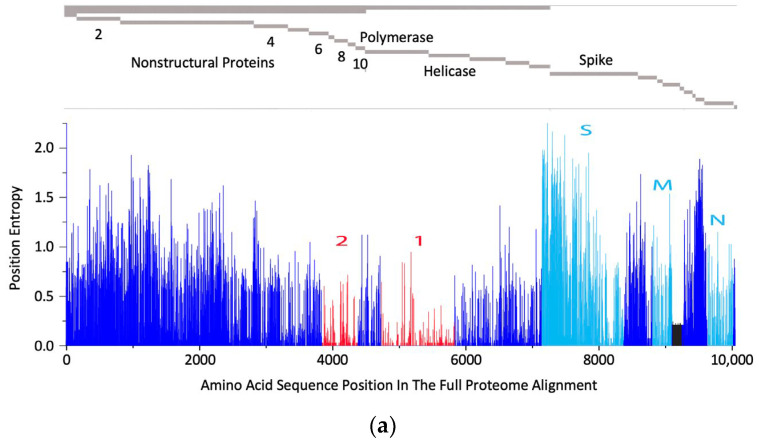

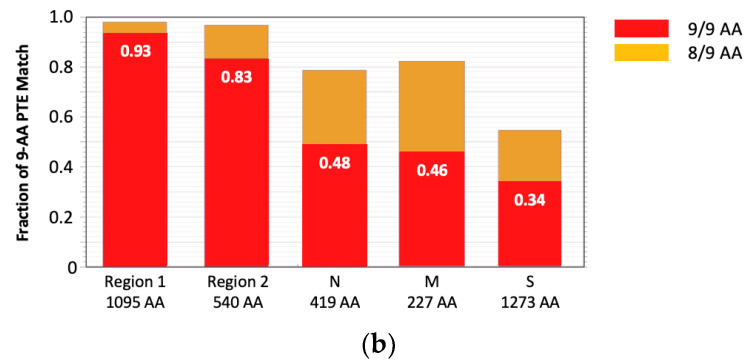
The COVconsv12 immunogen design. (**a**) The top diagram is a schematic representation of the sarbecovirus proteome with some of the key proteins named. The bottom graph shows the amino acid entropy across the entire sarbecovirus proteome. Red—the two continuous conserved protein regions 1 and 2 used to assemble the COVconsv12 immunogen; cyan—the spike (S), matrix (M) and nucleocapsid (N) proteins. (**b**) PTE coverage of sarbecovirus diversity by the COVconsv12 regions and selected proteins. Red 9/9 amino acid match; orange—8/9 amino acid match. The ancestral SARS-CoV-2 (Wuhan-WIV04-2019-EPI_ISL_402124) was the only representative of SAR-CoV-2 included in this summary of PTE conservation, and the Urbani variant SARS-CoV-1 (hSARS-CoV-AY278741.1-hACE2-Urbani-human-2003) related SARS-CoV-1 viruses, excluding the other members of these highly related viruses to avoid bias.

The rationale behind the pan-sarbecovirus T-cell immunogen design was described in detail by Dagotto et al. [11]. Briefly, the central theorem of our strategy is to focus vaccine-elicited CD8^+^ T cells on the most conserved (shared) regions of the sarbecovirus non-spike proteome so that the T-cell vaccines can be used together with the currently licensed spike vaccines and extend the spike vaccine-induced cross-reactive protection. Dalgotto et al. designated the conserved region used in their immunogen COV.Consv as region 1 [11], which included the RNA-dependent RNA polymerase (RdRp) and helicase protein parts contiguously expressed as part of open reading frame (Orf) 1ab (amino acids 4692–5785 of Orf 1ab, or 300–932 of RdRp and 1–461 of helicase). Our immunogen is called COVconsv12 and extends region 1 with the polymerase enzymatic site inactivated by mutating two aspartic acids to alanines [42] by region 2, which is derived from conserved non-structural proteins 6–10 (amino acids 3848–4385 of Orf 1a) (Figure 1a). A monoclonal mAb tag Pk was added to the C-terminus of the COVconsv12 protein to facilitate the gene product detection [35]. The whole protein immunogen contains 1642 amino acids (Figure S3). The COVconsv12 relevance for killer T-cell responses was determined by using the concept of potential T-cell epitopes (PTE), whereby a 9-amino acid-long window, the most frequent size of a CD8^+^ T-cell epitope, is slid across proteins by 1 amino acid at a time to determine, in each position, the vaccine proportional match to the sequences of the input viruses. This PTE analysis confirmed that COVconsv12 regions 1 and 2 were each much more conserved across sarbecoviruses than any of the spike, nucleocapsid, and matrix proteins (Figure 1b).

**Figure 2:**
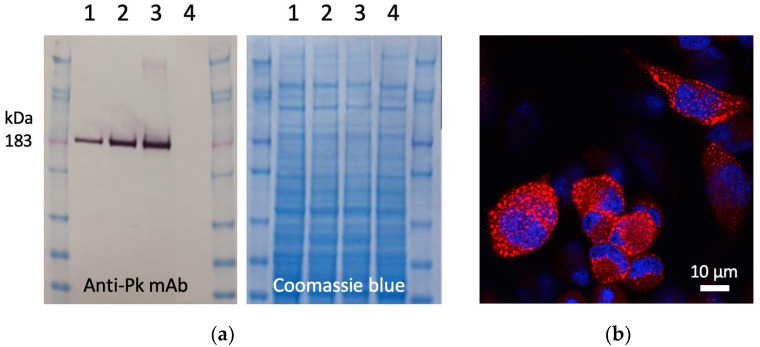
Expression of the *COVconsv12* transgene product in human cells. (**a**) Western blot and Coomassie blue-stained gel analysis of separated lysates of HeLa cells infected with ChAdOx1.COVconsv12 at MOI 10 (lanes 1), 50 (lanes 2), and 100 (lanes 3) for 1 day or uninfected (lanes 4). On the Western blot, the COVconsv12 protein was detected using C-terminal tag Pk (also known as SV5) recognized by mAb. The predicted relative molecular mass of COVconsv12 is indicated. (**b**) Confocal microscope analysis of HeLa cells infected at MOI 10 for 1 day using the anti-Pk mAb (red) to detect the COVconsv12 protein. The cell nuclei were stained with DAPI (blue).

To construct the ChAdOx1.COVconsv12 vaccine, a synthetic DNA fragment containing the *COVconsv12* open-reading frame was inserted into the E1 locus of the ChAdOx1 vector genome, a working virus stock was prepared, and the *COVconsv12* transgene product expression was readily detected in infected human cells (Figure 2).

### 3.2. Induction of Broad T-Cell Responses in Mice

**Figure 3:**
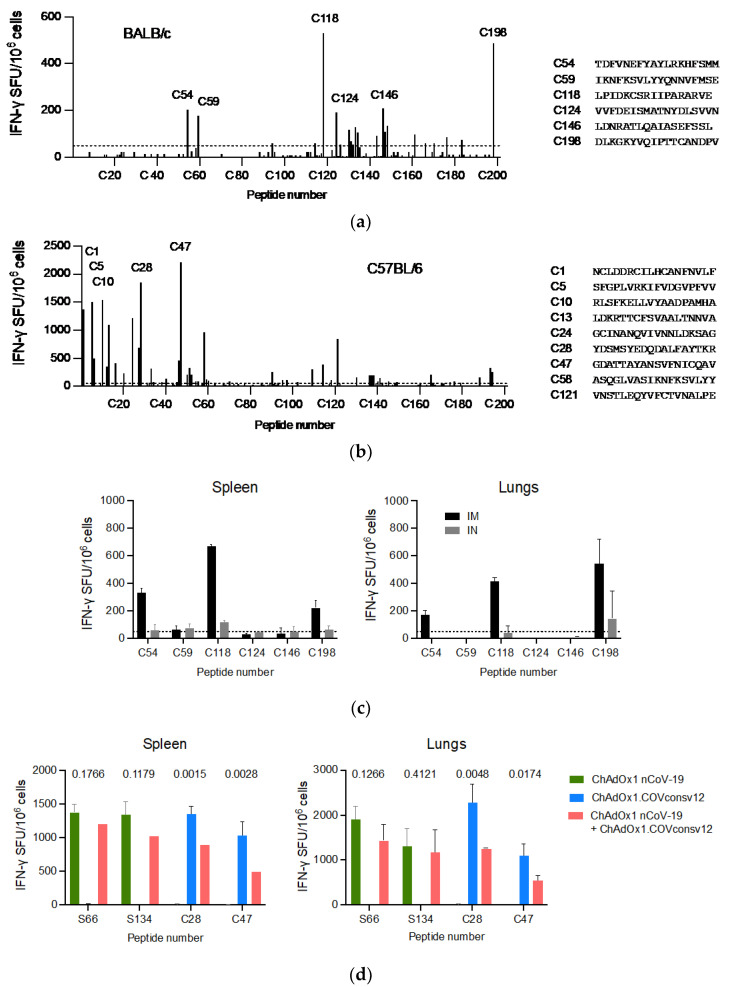
ChAdOx1.COVconsv12 mouse immunogenicity. Groups of mice were vaccinated once with ChAdOx1.COVconsv12 IM and their T-cell responses were assessed in an IFN-γ ELISPOT assay 9 days later. Using 201 individual 18-mer peptides overlapping by 11 amino acids spanning the entire length of the COVconsv12 immunogen, individual stimulatory peptides in the spleen of the BALB/c (**a**) and C56BL/6 (**b**) mice were determined, the strongest of which are shown next to the graph. (**c**) BALB/c mice received ChAdOx1.COVconsv12 IM (black) or IN (grey) and their vaccine-elicited T-cells against immunodominant peptides were enumerated in the spleens and lungs. (**d**) Frequencies of vaccine-elicited responses following IM coadministration of the ChAdOx1.COVconsv12 and ChAdOx1 nCoV-19 (spike) vaccines to the C57BL/6 mice. Two-tailed *p* values above compare frequencies to the same peptide using the Student’s *t*-test. S66 and S134—spike peptides; C28 and C47—COVconsv12 peptides. Median (IQR) responses are shown (n = 3 per group).

A well-defined mouse model was first used to confirm the ChAdOx1.COVconsv12 vaccine immunogenicity. BALB/c and C57BL/6 mice received a single vaccine dose intramuscularly (IM) and their vaccine-elicited T-cells were enumerated 9 days later. Stimulation with overlapping peptides across the entire immunogen in an IFN-*γ* ELISPOT assay revealed broadly specific T-cell responses in both murine strains (Figure 3a,b). The ChAdOx1.COVconsv12 vaccine was then delivered by the IM and intranasal (IN) routes to demonstrate induction of COVconsv12 T cells in the spleens and lungs (Figure 3c). Finally, the ChAdOx1.COVconsv12 vaccine was co-administered with the spike vaccine ChAdOx1 nCoV-19. This elicited parallel responses to COVconsv12 and the spike, although the mixed immunization resulted in lower specific T-cell frequencies compared to the individual vaccines alone. The difference reached statistical significance for the COVconsv12-derived peptides (Figure 3d). Overall, the murine experiments demonstrated induction of broad coronavirus-specific T-cell responses.

### 3.3. Hints of Improved Recovery after Virus Challenge through Combination of Spike and T-Cell Vaccines in Hamsters

Infection of Syrian hamsters with SARS-CoV-2 results in the development of a robust upper and lower respiratory tract disease [43], which was used for the preclinical efficacy testing of several COVID-19 vaccines including Oxford-AstraZeneca ChAdOx1 nCoV-19 [1,3,44]. Here, we first vaccinated two male and two female Syrian hamsters with 2.5 × 10^8^ vp of ChAdOx1.COVconsv12 and assayed the splenocytes, lung cells, and peripheral blood mononuclear cells for vaccine-induced coronavirus-specific T cells 13 days later. IFN-γ-producing SFUs were detected upon peptide pool and individual peptide restimulations reaching at best approximately 500 SFU/10^6^ cells, although likely background responses were observed, and the more positive peptide specificities were not entirely consistent between tissues. Discrepancies were also detected between the pool and single peptide responses (Figure S4).

**Figure 4:**
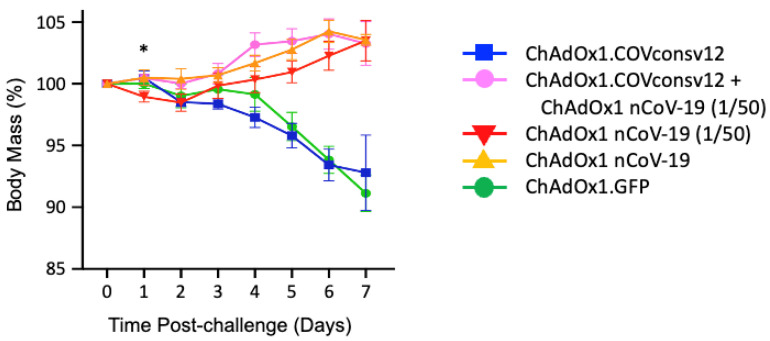
Post-challenge hamster body mass changes. Day 0 represents the day of the challenge. Data are shown as the mean ± SEM (n = 5 per group). *—two-tailed *p* = 0.023 using Student’s *t*-test ChAOx1.COVconsv12 + ChAdOx1 nCoV-19 (1/50) vs. ChAdOx1 nCoV-19 (1/50) alone.

Next, five groups of five mixed male/female Syrian hamsters were immunized with the ChAdOx1.COVconsv12 vaccine alone, ChAdOx1.COVconsv12 together with 1/50th of the protective spike vaccine ChAdOx1 nCoV-19 (ChAdOx1 nCoV-19 (1/50)), ChAdOx1 nCoV-19 (1/50), full hamster-protective dose of ChAdOx1 nCoV-19 alone, and a ChAdOx1 vector with irrelevant immunogen green fluorescent protein (GFP) as a negative control (Table 1). The animals were challenged with SARS-CoV-2, strain Victoria-1, 2 weeks later (see Figure S5 for the alignment of COVconsv12 and the challenge virus), and their body mass was recorded daily thereafter. The ChAdOx1 nCoV-19 protective dose, ChAdOx1 nCoV-19 (1/50), and the combined regimen allowed animals to gain body mass steadily after 2 days post-challenge. There was a statistically significant difference in the body mass between animals receiving the ChAdOx1.COVconsv12 and ChAdOx1 nCoV-19 (1/50) together (Group 2) and ChAdOx1 nCoV-19 (1/50) only (Group 3) on day 1 after challenge (two-tailed *p* = 0.023; Student’s *t*-test). While this trend was maintained until day 6, the difference was no longer significant. The ChAdOx1.COVconsv12 vaccine alone (Group 1) offered no benefit over the first 7 days after the coronavirus challenge, and the hamsters reacted similarly to the negative control-treated animals (Group 5), leading to a loss of about 7% of their body mass (Figure 4).

**Figure 5:**
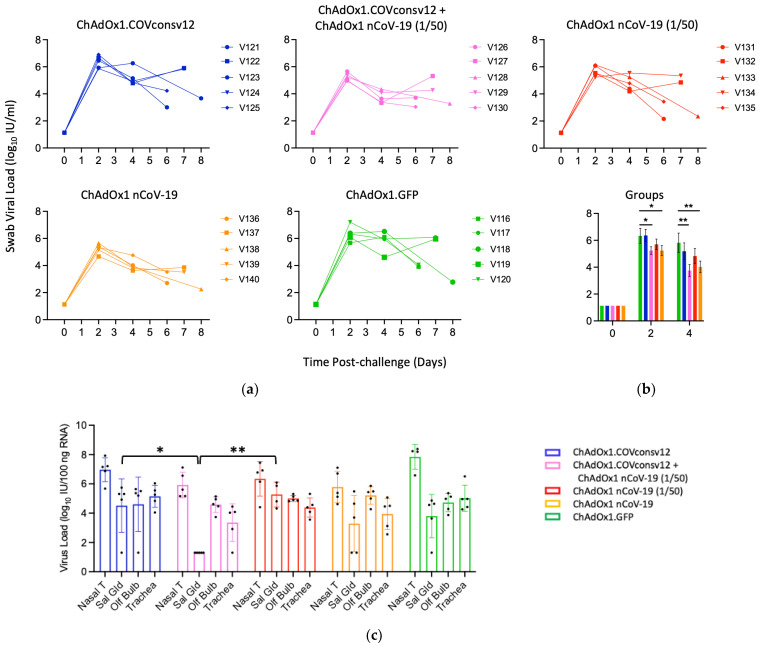
Virus load analyses after challenge. (**a**,**b**) Swab and (**c**) tissue virus loads were determined by qPCR. The detection limit was the virus load of 0.1 genome copies per 100 ng of RNA. Syrian hamsters V121-V123, V126, V127, V131-V133, V136, V137, V116, and V117 were males and V124, V125, V128-V130, V134, V135, V138-V140, and V118-V120 were females. One or two animals from each group were euthanized daily from day 5 post-challenge to facilitate optimal sample management. (**b**) Mean ± SEM of virus load is shown for individual groups. A Student’s *t*-test was used for individual comparisons with *p* values adjusted using a Benjamini–Hochberg procedure [45]. (**c**) Nasal T—nasal tissue; Sal Gld—salivary gland; Olf Bulb—olfactory bulb. *—0.01 < *p* ≤ 0.05; **—*p* ≤ 0.01.

Viral load in the upper respiratory tract was measured by RT-qPCR of SARS-CoV-2 in oral swabs. The combined ChAdOx1.COVconsv12 + ChAdOx1 nCoV-19 (1/50) regimen and the spike vaccine alone were significantly more efficient in reducing the virus load compared to the other three vaccinations, including the ChAdOx1 nCoV-19 (1/50) alone on day 4 (*p* = 0.010; two-tailed Student’s *t*-test Groups 2 vs. 3) (Figure 5a,b). The overall pattern of the relative virus distribution and the number of detected genome copies across the tested tissues broadly concurred with the swab virus loads showing a trend of the lowest viremia achieved by the ChAdOx1 nCoV-19 and the combined ChAdOx1.COVconsv12 and ChAdOx1 nCoV-19 (1/50) vaccination (Figure 5c).

**Figure 6:**
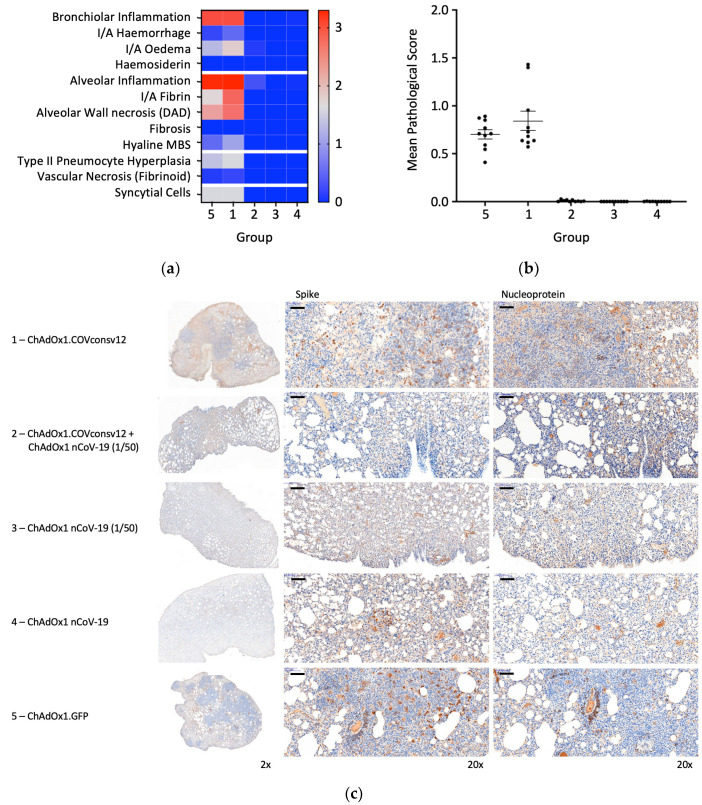
Lung pathology. (**a**) Heatmap of the mean group pathology score for each parameter assessed. (**b**) Mean lung pathological score calculated as the sum of individual parameter scores multiplied by the % of tissue affected for each individual’s left lobe or right cranial lobe. (**c**) Immunohistochemical staining for SARS-CoV-2 spike or nucleoprotein (NP) in hamster lungs 6 days post-challenge. Low and high-resolution images (scale bars represent 100 µm).

Randomly selected animals in all groups were examined post-mortem for overt signs of pathology. The negative control animals, which received ChAdOx1.GFP, showed pathology expected in the hamster model following the SARS-CoV-2 challenge. A very similar pathology was observed in the ChAdOx1.COVconsv12 group. In contrast, vaccination with the spike vaccine ChAdOx1 nCoV-19 ameliorated such pathological changes (Figure 6a,b). This was further supported by the immunohistochemical staining of the lung sections for the challenge virus spike and nucleoprotein, noticeable bronchiolar and alveolar eosinophil inflammation, vacuolization, and more clumps of positively stained syncytial cells in the absence of the spike vaccine (Figure 6c). Thus, in the Syrian hamster model, only marginal signs of improved recovery were detected following the addition of the T-cell vaccine. No significant differences between males and females were detected.

## 4. Discussion

In the present work, we describe the design of a new candidate pan-sarbecovirus T-cell immunogen delivered by a purposed-designed vaccine vector assembled into vaccine ChAdOx1.COVconsv12. The *COVconsv12* synthetic transgene was inserted into a genome of in-human proven simian adenovirus vector ChAdOx1 and the expression of its product was confirmed in infected human cells. Intramuscular administration of the ChAdOx1.COVconsv12 vaccine to three rodent models resulted in no adverse events. Vaccination of the BALB/c and C57BL/6 mice induced broad T-cell responses. In outbred Syrian hamsters, coronavirus-specific T cells were detected using a commercially available kit, though their specificity was somewhat less consistent, likely due to less well-established reagents in this model (Figure S4). Vaccination of hamsters with the T-cell vaccine alone provided no detectable protective immunity against the experimental challenge with the Victoria-1 strain of SARS-CoV-2. A moderate additional beneficial contribution of the ChAdOx1.COVconsv12 vaccine-elicited T-cells to the protection against the challenge virus was detected when 1/50th of the protective dose of the Oxford-AstraZeneca ChAdOx1 nCoV-19 vaccine was used for vaccination. Overall, these results concur with the protective efficacy of the RhAd52.CoV.Consv vaccine in mice [11] and together demonstrate the proof of concept that vaccines including only the most highly conserved regions of the sarbecovirus proteome can elicit CD8^+^ T-cell responses contributing to protection.

The present work has several limitations. The study would benefit from a longer-term follow-up of the challenged animals and the vaccine effects beyond 7 days. Also, antibodies that could have been induced to the immunogen subunits could have contributed to the protective effect of the ChAdOx1.COVconsv12 vaccination. While this remains a possibility worthy of investigation, antibodies to internal non-structural viral proteins rarely contribute to antiviral effects due to access issues. Finally, the role of innate responses and cytokines were not addressed in this work because these are determined predominantly by the much more complex vaccine vector rather than by the nature of the transgene product. Furthermore, innate responses are very different in hamsters from humans, and thus, such studies are warranted in the latter relevant species.

The COVconsv12 immunogen has the potential to be developed further towards human testing because, ultimately, it only matters what happens in people. The PTE coverage and, thus, the relevance for T-cell induction of these conserved regions to other more distant coronavirus lineages could be broadened by including a polyvalent cocktail of Epigraphs [27,46]. We and others have used the Epigraph computer algorithm to design such mutually complementing immunogen cocktails to extend the breadth of immune responses for a pan-filovirus vaccine [47] and broad T-cell and B-cell responses against the influenza virus [48,49,50], both of which conferred protection in animal models against highly diverse viral challenges. Furthermore, unlike the single vaccine-dose protection against SARS-CoV-2 achieved by several vaccines important for rapid antibody-based protection of the public at the start of a new outbreak, induction of more protective T cells requires a heterologous vaccine boost, such as that delivered by the poxvirus MVA vector [51,52], to achieve higher frequencies of relevant CD8^+^ T cells and a more beneficial protective effect. In essence, broadly specific killer T cells elicited by vaccination before an outbreak can slow new emerging viruses, lessen disease severity, and buy time for the more tailored spike vaccines or other interventions to be manufactured and deployed. Effective T cells show greater durability than the spike-specific antibodies [16,53,54,55] and may further fortify antibody protection. Through their association with better clinical outcomes, CD8^+^ T cells may help to prevent long COVID-19 [56,57].

## 5. Conclusions

The rapid development and deployment of the COVID-19 vaccines was a great success in biomedical research, which saved many millions of lives. The mechanisms contributing to the containment of the SARS-CoV-2 infection and amelioration of the COVID-19 disease were multifactorial. Their deeper understanding will guide the optimization of the next-generation vaccines against ever-emerging new coronavirus variants.

## Figures and Tables

**Table 1:** Syrian Hamster Vaccination Groups.

Grp	Sex (M/F) ^1^	Vaccine (Dose)
1	3/2	ChAdOx1.COVconsv12 (2.5 × 10^8^ vp)
2	2/3	ChAdOx1.COVconsv12 (2.5 × 10^8^ vp) + ChAdOx1 nCoV-19 (1/50) (5 × 10^6^ vp) ^2^
3	3/2	ChAdOx1 nCoV-19 (1/50) (5 × 10^6^ vp) ^2^
4	2/3	ChAdOx1 nCoV-19 (2.5 × 10^8^ vp) ^3^
5	2/3	ChAdOx1.GFP (2.5 × 10^8^ vp)

^1^ M/F—male/female. ^2^ 1/50th of the protective hamster dose of the ChAdOx1 nCoV-19 vaccine. ^3^ Proven protective hamster dose of the ChAdOx1 nCoV-19 vaccine [1].

## Data Availability

All data are provided within the manuscript.

## Supplementary Materials

The following supporting information can be downloaded at: https://www.mdpi.com/article/10.3390/vaccines12090965/s1, Figure S1: 205 sarbecoviruses used for bioinformatical analysis. Figure S2: Maximum likelihood phylogenetic tree based on an alignment of the full proteome. Figure S3: Amino acid sequence of the COVconsv12 immunogen. Figure S4: Pilot immunogenicity study of ChAdOx1.COVconsv12 in Syrian hamsters. Figure S5: Amino acid alignment of the challenge virus and the vaccine.
