# Supplementary material for: Design, Immunogenicity and Preclinical Efficacy of the ChAdOx1.COVconsv12 Pan-Sarbecovirus T-Cell Vaccine
Source: Vaccines (Basel). 2024 Aug 26;12(9):965. doi: 10.3390/vaccines12090965 (PMC11436245; doi:10.3390/vaccines12090965)
Supplement: Supplementary file 1 [file vaccines-12-00965-s001.zip › vaccines-3156622-supplementary.pdf]

Extended name of the viral sequence	GISAID (EPI_ISL) or GenBank accession numbers	The subset of viruses used to create Figure 1B
hCoV-19-Wuhan-WIV04-2019-EPI_ISL_402124-2019-12-30	EPI_ISL_402124	x
hSARS-CoV-AY278741.1-hACE2-Urbani-human-2003	AY278741.1	x
hSARS-CoV-AY313906-hAce2-GD69-human-x-GuangdongChina	AY313906	
hSARS-CoV-AY291451-hAce2-TW1-human-2003-Taiwan	AY291451	
hSARS-CoV-AY282752-hAce2-CUHK-Su10-human-2003-HongKongChina	AY282752	
hSARS-CoV-AY4852771-hAce2-Sino1-11-human-2003-China	AY4852771	
hSARS-CoV-AY559088-hAce2-SinP1-human-x-Singapore	AY559088	
hSARS-CoV-AY461660-hAce2-SoD-human-x-Russia	AY461660	
hSARS-CoV-AY283795-hAce2-Sin2677-human-x-Singapore	AY283795	
hSARS-CoV-hAce2-NC004718-human-2003-Canada	NC004718	
hSARS-CoV-DQ182595-hAce2-ZJ0301-human-2003-HangzhouChina	DQ182595	
hSARS-CoV-AY559084-hAce2-Sin3765V-human-x-Singapore	AY559084	
hSARS-CoV-FJ882963-hAce2-P2-2004-hum-USA	FJ882963	
hSARS-CoV-AY463059-hAce2-ShanghaiQXC1-human-2003-2004-ShanghaiChina	AY463059	
hSARS-CoV-AY5087241-hAce2-NS-1-human-2003-NingxiaChina	AY5087241	
hSARS-CoV-AY394994-hAce2-HSZ-Bc-human-x-x	AY394994	
hSARS-CoV-AY394995-hAce2-HSZ-Cc-human-x-x	AY394995	
hSARS-CoV-AY613947-hAce2-GZ0402-human-x-China	AY613947	
Civet-AY545918-x-HCgZ3203-x-Pagumalarvata-China	AY545918	x
Civet-AY572038-x-civet020-2004-Pagumalarvata-GuangzhouChina	AY572038	
Civet-AY515512-x-HCgZ6103-x-Pagumalarvata-China	AY515512	
Civet-AY545914-x-HCgZ7903-x-Pagumalarvata-ShenzenChina	AY545914	
Civet-AY304488-x-SZ16-Pagumalarvata-2003-HongKongChina	AY304488	
Civet-AY304486-hAce2-SZ3-Pagumalarvata-2003-GuangdongChina	AY304486	
hSARS-CoV-AY3905561-hAce2-GZ02-human-2003-GuangzhouChina	AY3905561	
hSARS-CoV-AY2784892-hAce2-GD01-human-2003-GuangdongChina	AY2784892	
hSARS-CoV-AY395003-hAce2-ZS-C-human-x-x	AY395003	
Bat-OK017852-x-YN2020B-Rhsinicus-2020-YunnanChina	OK017852	x
Bat-OK017855-x-YN2020E-Rhsinicus-2020-YunnanChina	OK017855	x
Bat-OK017856-x-YN2020F-Rhsinicus-2020-YunnanChina	OK017856	x
Bat-OK017857-x-YN2020G-Rhsinicus-2020-YunnanChina	OK017857	x
Bat-OK017853-x-YN2020C-Rhsinicus-2020-YunnanChina	OK017853	x
Bat-OK017854-x-YN2020D-Rhsinicus-2020-YunnanChina	OK017854	x
Bat-KY417146-hAce2-Rs4231-Rhsinicus-2013-YunnanChina	KY417146	x
Bat-KY417150-hAce2-Rs4874-Rhsinicus-2013-YunnanChina	KY417150	x
Bat-KT444582-hAce2-WIV16-Rhsinicus-2013-YunnanChina	KT444582	x
Bat-OK017858-x-YN2020H-Rhsinicus-2020-YunnanChina	OK017858	x
Bat-KC881005-hAce2-RsSHC014-Rhsinicus-2012-YunnanChina	KC881005	x
Bat-KY417144-hAce2-Rs4084-Rhsinicus-2012-YunnanChina	KY417144	x
Bat-KC881006-x-Rs3367-Rhsinicus-2012-YunnanChina	KC881006	x
Bat-KF367457-hAce2-WIV1-Rhsinicus-2012-YunnanChina	KF367457	x
Bat-KY417152-x-Rs9401-Rhsinicus-2015-YunnanChina	KY417152	x
Bat-KY417151-hAce2-Rs7327-Rhsinicus-2014-YunnanChina	KY417151	x
Bat-MK211376-x-YN2018B-Rh-affinis-2016-YunnanChina	MK211376	x
Bat-OK017849-x-YN2016C-Rhsinicus-2016-YunnanChina	OK017849	x
Bat-OK017850-x-YN2016D-Rhsinicus-2016-YunnanChina	OK017850	x
Bat-OK017851-x-YN2016E-Rhsinicus-2016-YunnanChina	OK017851	x
Bat-OK017848-x-YN2016B-Rhsinicus-2016-YunnanChina	OK017848	x
Bat-OK017847-x-YN2016A-Rhsinicus-2016-YunnanChina	OK017847	x
Bat-KP886808-x-YNLF31C-Rhsinicus-2013YunnanChina	KP886808	x
Bat-KP886809-x-YNLF34C-Rhsinicus-2013-YunnanChina	KP886809	x
Bat-KY417145-nohAce2-Rf4092-Rhferrumequinum-2012-YunnanChina	KY417145	x
Bat-KU973692-x-F46-Rhpusillus-2012-YunnanChina	KU973692	x
Bat-KJ473816-nohAce2-YN2013-Rhsinicus-2013-YunnanChina	KJ473816	x
Bat-KY770858-x-Anlong103-Rhsinicus-2013-GuizhouChina	KY770858	x
Bat-KY770859-x-Anlong-112-Rhsinicus-2013-GuizhouChina	KY770859	x
Bat-FJ588686-x-Rs672-Rhsinicus-GuizhouChina	FJ588686	x
Bat-KY417143-nohAce2-Rs4081-Rhsinicus-2012-YunnanChina	KY417143	x
Bat-KY417149-x-Rs4255-Rhsinicus-2013-YunnanChina	KY417149	x
Bat-MK211378-x-YN2018D-Rhaffinis-2018-YunnanChina	MK211378	x
Bat-KY417142-nohAce2-As6526-Aselliscusstoliczkanus-2014-YunnanChina	KY417142	x

Figure S1. 205 sarbecovirus sequences used for bioinformatical analysis.

Bat-MK211377-x-YN2018C-Rhaffinis-2018-YunnanChina	MK211377	x
Bat-KY417147-nohAce2-Rs4237-Rhsinicus-2013-YunnanChina	KY417147	x
Bat-KY417145-nohAce2-Rs4247-Rhsinicus-2013-YunnanChima	KY417145	x
Bat-MK211375-x-YN2018A-Rhaffinis-2018-YunnanChina	MK211375	x
Bat-OK017793-x-YN2020A-Rhaffinis-2020-YunnanChina	OK017793	x
Bat-OK017792-x-GD2017F-Rhaffinis-2017-GuangdongChina	OK017792	x
Bat-DQ071615-nohAce2-Rp3-Rhpearsonii-2004-GuangxiChina	DQ071615	x
Bat-OK017829-x-GZ2021C-Rhsinicus-2021-GuizhouChina	OK017829	x
Bat-OK017831-x-GZ2021I-Rhsinicus-2021-GuizhouChina	OK017831	x
Bat-OK017830-x-GZ2021H-Rhsinicus-2021-GuizhouChina	OK017830	x
Bat-KJ473815-nohAce2-GX2013-Rhsinicus-2013-GuangxiChina	KJ473815	x
Bat-OK017842-x-JX2021M-Rhsinicus-2021-JiangxiChina	OK017842	x
Bat-OK017843-x-JX2021N-Rhsinicus-2021-JiangxiChina	OK017843	x
Bat-OK017841-x-JX2021L-Rhsinicus-2021-JiangxiChina	OK017841	x
Bat-OK017838-x-JX2021G-Rhsinicus-2021-JiangxiChina	OK017838	x
Bat-OK017840-x-JX2021K-Rhsinicus-2021-JiangxiChina	OK017840	x
Bat-OK017839-x-JX2021J-Rhsinicus-2021-JiangxiChina	OK017839	x
Bat-OK017844-x-JX2021O-Rhsinicus-2021-JiangxiChina	OK017844	x
Bat-OK017845-x-JX2021P-Rhsinicus-2021-JiangxiChina	OK017845	x
Bat-OK017808-x-FJ2021A-Rhsinicus-2021-FujianChina	OK017808	x
Bat-OK017809-x-FJ2021D-Rhsinicus-2021-FujianChina	OK017809	x
Bat-OK017811-x-FJ2021M-Rhsinicus-2021-FujianChina	OK017811	x
Bat-OK017810-x-FJ2021E-Rhsinicus-2021-FujianChina	OK017810	x
Bat-OK017807-x-AH2021A-Rhsinicus-2021-AnhuiChina	OK017807	x
Bat-OK017837-x-JX2021C-Rhsinicus-2021-JiangxiChina	OK017837	x
Bat-OK017802-x-HB2020E-Rhspp-2020-HubeiChina	OK017802	x
Bat-OK017801-x-HB2020D-Rhspp-2020-HubeiChina	OK017801	x
Bat-OK017836-x-JX2021AC-Rhsinicus-2021-JiangxiChina	OK017836	x
Bat-GQ153542-x-HKU3-7-Rhsinicus-2006-GuangdongChina	GQ153542	x
Bat-GQ1535431-x-Bat-SARS-coronavirus-HKU3-8	GQ1535431	x
Bat-GQ153547-x-HKU3-12-Rhsinicus-2007-05-15	GQ153547	x
Bat-DQ0841991-x-HKU3-2b-Rhsinicus-2005-HongKongChina	DQ0841991	x
Bat-GQ1535451-x-HKU3-10-Rhsinicus-2006-HongKongChina	GQ1535451	x
Bat-GQ1535481-nohAce2-HKU3-13b-Rhsinicus-2007-HongKongChina	GQ1535481	x
Bat-DQ022305-x-HKU3-3-Rhsinicus-2005-HongKongChina	DQ022305	x
Bat-DQ022305-x-HKU31-Rhsinicus-2005-HongKongChina	DQ022305	x
Bat-FJ211859-x-Consensus-BatSCOV-x-x-x	FJ211859	x
Bat-OK017812-x-GD2016B-Rhsinicus-2016-GuangdongChina	OK017812	x
Bat-OK017813-x-GD2017G-Rhsinicus-2017-GuangdongChina	OK017813	x
Bat-OK017819-x-GD2017M-Rhsinicus-2017-GuangdongChina	OK017819	x
Bat-OK017815-x-GD2017I-Rhsinicus-2017-GuangdongChina	OK017815	x
Bat-OK017814-x-GD2017H-Rhsinicus-2017-GuangdongChina	OK017814	x
Bat-OK017821-x-GD2017O-Rhsinicus-2017-GuangdongChina	OK017821	x
Bat-OK017820-x-GD2017N-Rhsinicus-2017-GuangdongChina	OK017820	x
Bat-OK017817-x-GD2017K-Rhsinicus-2017-GuangdongChina	OK017817	x
Bat-OK017816-x-GD2017J-Rhsinicus-2017-GuangdongChina	OK017816	x
Bat-OK017818-x-GD2017L-Rhsinicus-2017-GuangdongChina	OK017818	x
Bat-OK017824-x-GD2017W-Rhsinicus-2017-GuangdongChina	OK017824	x
Bat-OK017822-x-GD2017P-Rhsinicus-2017-GuangdongChina	OK017822	x
Bat-OK017823-x-GD2017Q-Rhsinicus-2017-GuangdongChina	OK017823	x
Bat-OK017826-x-GD2019B-Rhsinicus-2019-GuangdongChina	OK017826	x
Bat-OK017825-x-GD2019A-Rhsinicus-2019-GuangdongChina	OK017825	x
Bat-OK017827-x-GD2019D-Rhsinicus-2019-GuangdongChina	OK017827	x
Bat-OK017828-x-GD2019E-Rhsinicus-2019-GuangdongChina	OK017828	x
Bat-KF569996-hAce2-LYRa11-Rhaffinis-YunnanChina	KF569996	x
Bat-EPI_ISL_1699447-x-RmYN07-Rhmalayanus-2020-YunnanChina	EPI_ISL_1699447	x
Bat-EPI_ISL_1699443-x-RsYN03-Rhsinicus-2019-YunnanChina	EPI_ISL_1699443	x
Bat-JX993988-nohAce2-Yunnan2011-Chaerephonplicatus-2011-YunnanChina	JX993988	x
Bat-EPI_ISL_1699449-x-RsYN09-Rhstheno-2020-YunnanChina	EPI_ISL_1699449	x
Bat-MK211374-x-SC2018-Rhsp-2018-SichuanChina	MK211374	x
Bat-KJ473814-nohAce2-SL-HuB2013-Rhsinicus-2013-HubeiChina	KJ473814	x
Bat-OK017835-x-HN2021F-Rhsinicus-2021-HunanChina	OK017835	x
Bat-OK017834-x-HN2021E-Rhsinicus-2021-HunanChina	OK017834	x
Bat-DQ648857-nohAce2-SL-CoV279-Rhmacrotis-2004-HubeiChina	DQ648857	x

Figure S1. 205 sarbecovirus sequences used for bioinformatical analysis. Continued.

Bat-DQ412043-x-Rm1-Rhmacrotis-2004-HubeiChina	DQ412043	x
Bat-OK017860-x-JX2021D-Rhsiamensis-2021-JiangxiChina	OK017860	x
Bat-OK017833-x-HN2021D-Rhsinicus-2021-HunanChina	OK017833	x
Bat-OK017832-x-HN2021C-Rhsinicus-2021-HunanChina	OK017832	x
Bat-OK017859-x-GX2019A-Rhsiamensis-2019-GuangxiChina	OK017859	x
Bat-JX993987-nohAce2-Shaanxi2011-Rhpusillus.2011-ShaanxiChina	JX993987	x
Bat-OK017846-x-SC2018B-Rhsinicus-2016-SichuanChina	OK017846	x
Bat-OM240725.1-RaCH025-no-R.affinis-2020-ChinaGuangdong	OM240725.1	x
Bat-DQ648856-nohAce2-BtCoV273-Rhferrumequinum-2004-HubeiChina	DQ648856	x
Bat-DQ412042-nohAce2-Rf1-Rhferrumequinum-2004-HubeiChina	DQ412042	x
Bat-KY770860-x-Jiyuan84-Rhferrumequinum-2012-HenanChina	KY770860	x
Bat-KJ473812-nohAce2-HeB2013-Rhferrumequinum2013-HebeiChina	KJ473812	x
Bat-KJ473813-x-SX2013-Rhferrumequinum2013-ShanxiChina	KJ473813	x
Bat-ON378802.1-BatCoV_B20-50-x-2020-SouthKorea	ON378802.1	x
Bat-KJ473811-nohAce2-Rhferrumequinum-JL2012-2012-JilinChina	KJ473811	x
Bat-OK017794-x-LN2020A-Rhferrumequinum-2020-LiaoningChina	OK017794	x
Bat-OK017799-x-LN2020G-Rhferrumequinum-2020-LiaoningChina	OK017799	x
Bat-OK017797-x-LN2020E-Rhferrumequinum-2020-LiaoningChina	OK017797	x
Bat-OK017795-x-LN2020B-Rhferrumequinum-2020-LiaoningChina	OK017795	x
Bat-OK017798-x-LN2020F-Rhferrumequinum-2020-LiaoningChina	OK017798	x
Bat-OK017800-x-LN2020H-Rhferrumequinum-2020-LiaoningChina	OK017800	x
Bat-MZ190138-x-Khosta-2-Rhferrumequinum-2020-Russia	MZ190138	x
Bat-MW719567-x-RhGB01-2020-Rhhipposideros-UK	MW719567	x
Bat-MZ190137-x-Khosta-1-Rhferrumequinum-2020-Russia	MZ190137	x
Bat-GU190215-nohAce2-BM48-31-Rhblasii-2008-Bulgaria	GU190215	x
Bat-KY352407-x-BtKY72-Rhsp-2007-Kenya	KY352407	x
Bat-MT726043-nohAce2-PDF-2386-Rhsp-2013-Uganda	MT726043	x
Bat-MT726044-nohAce2-PDF-2370-Rhsp-2013-Uganda	MT726044	x
Bat-MT726043-nohAce2-PRD-0038-Rhsp-2010-Rwanda	MT726043	x
hCoV19-WA.1-MN985325.1-ACE2-SARS-CoV-2/human/USA/WA-CDC-02982586-0(	MN985325.1	
hCoV19-hum-hAce2-Mu-B1621--EPI_ISL_2086281-2021-03-29-B1621-USANew-Jer	EPI_ISL_2086281	
hCoV19-hum-hAce2-Beta-B1351--EPI_ISL_940877-2020-11-12-B1351-South-Africa	EPI_ISL_940877	
hCoV19-hum-hAce2-Iota-B1526--EPI_ISL_1300881-2020-12-06-B1526-USANew-Yc	EPI_ISL_1300881	
hCoV19-hum-hAce2-Epsilon-B1429--EPI_ISL_648527-2020-10-16-B1429-USACalifc	EPI_ISL_648527	
hCoV19-hum-hAce2-Eta-B1525--EPI_ISL_760883-2020-12-15-B1525-United-Kingdc	EPI_ISL_760883	
hCoV19-hum-hAce2-Alpha-B117--EPI_ISL_674612-2020-11-13-B117-United-Kingdc	EPI_ISL_674612	
hCoV19-hum-hAce2-Lambda-C37--EPI_ISL_1138413-2021-01-12-C37-PeruLima-LIN	EPI_ISL_1138413	
hCoV19-hum-hAce2-Zeta-P2--EPI_ISL_717936-2020-10-29-P2-BrazilRio-de-Janeiro	EPI_ISL_717936	
hCoV19-hum-hAce2-Theta-P3--EPI_ISL_1660475-2021-01-24-P3-Hong-Kong-HKU-2	EPI_ISL_1660475	
hCoV19-hum-hAce2-Gamma-P1--EPI_ISL_2777382-2020-12-03-P1-BrazilAmazona	EPI_ISL_2777382	
hCoV19-hum-hAce2-Kappa-B16171--EPI_ISL_1384866-2021-02-03-B16171-IndiaM	EPI_ISL_1384866	
hCoV19-hum-hAce2-Delta_AY.4__AY.4|EPI_ISL_1758376|United_Kingdom|2021-(	EPI_ISL_1758376	
hCoV19-hum-hAce2-Omicron-BA1--EPI_ISL_8185458.2021-11-29.BA.1.North-Amei	EPI_ISL_8185458	
hCoV19-hum-hAce2-Omicron-BA.3--EPI_ISL_9266834.2022-01-03.BA3.Europe-Pola	EPI_ISL_9266834	
hCoV19-hum-hAce2-Omicron-BA2--EPI_ISL_8135710.2021-12-13.BA.2.Europe-Den	EPI_ISL_8135710	
hCoV19-hum-hAce2-Omicron-BA.5--BA.5|EPI_ISL_11542604|South_Africa|2022-(	EPI_ISL_11542604	
hCoV19-hum-hAce2-Omicron-XBB.1.5-EPI_ISL_16763290-hAce2-USA/TX-CDC-LC09	EPI_ISL_16763290	
hCoV19-hum-hAce2-Omicron-JN.1/England/CLIMB-CM7YE7NN/2023|JN.1_EPI_ISI	EPI_ISL_18707986	
Bat-EPI_ISL_402131-hAce2-RaTG13-Rhaffinis-2013-YunnanChina	EPI_ISL_402131	x
Bat-EPI_ISL_4302644-hAce2-BANAL-20-52-2020-Rhmalayanus-2020-Laos	EPI_ISL_4302644	x
Bat-EPI_ISL_4302645-hAce2-BANAL-20-236-Rhpusillus-2020-Laos	EPI_ISL_4302645	x
Bat-EPI_ISL_4302647-hAce2-BANAL-20-103-Rhmarshalli-2020-Laos	EPI_ISL_4302647	x
Bat-EPI_ISL_412977-x-RmYN02-Rhmalayanus-2019-YunnanChina	EPI_ISL_412977	x
Bat-EPI_ISL_4302646-nohAce2-BANAL-20-116-Rhmalayanus-2020-Laos	EPI_ISL_4302646	x
Bat-EPI_ISL_4302648-nohAce2-BANAL-20-247-Rhmalayanus-2020-Laos	EPI_ISL_4302648	x
Bat-MW251308-x-RacCS203-Rhacuminatus-2020-Thailand	MW251308	x
Bat-EPI_ISL_1699446-x-RpYN06-2020-Rhpusillus-2020-YunnanChina	EPI_ISL_1699446	x
Bat-EPI_ISL_1098866-x-PrC31-Rhblythi-2018-YunnanChina	EPI_ISL_1098866	x
Bat-OK017806-x-YN2021-Rhpusillus-2021-YunnanChina	OK017806	x
Bat-OK017805-x-HN2021G-Rhpusillus-2021-HunanChina	OK017805	x
Bat-OK017803-x-HN2021A-Rhpusillus-2021-HunanChina	OK017803	x
Bat-OK017804-x-HN2021B-Rhpusillus-2021-HunanChina	OK017804	x
Bat-MG772934-nohAce2-ZXC21-Rhsinicus-2015-ZhejiangChina	MG772934	x
Bat-MG772933-nohAce2-ZC45-Rhsinicus-2017-ZhejiangChina	MG772933	x

Figure S1. 205 sarbecovirus sequences used for bioinformatical analysis. Continued.

Bat-EPI_ISL_852604-x-RShSTT182-2010-Cambodia	EPI_ISL_852604	x
Bat-EPI_ISL_852605-x-RShSTT200-2010-Cambodia	EPI_ISL_852605	x
Pango-EPI_ISL_410721-hAce2-GD12019-2019-GuangdongChina	EPI_ISL_410721	x
Pango-EPI_ISL_412860-x-x-SL-CoV-GD-P1La-2019-03-19	EPI_ISL_412860	x
Pango-EPI_ISL_410538-x-GX-P4L-Manis-javanica-2017-GuangxiChina	EPI_ISL_410538	x
Pango-EPI_ISL_410541-x-GX-P5E-EPI_ISL_410541-2017-GuangxiChina	EPI_ISL_410541	x
Pango-EPI_ISL_410542-hAce2-P2V-Manisjavanica-2017-GuangxiChina	EPI_ISL_410542	x
Pango-EPI_ISL_410540-x-GX-P5L-Manisjavanica-2017-GuangxiChina	EPI_ISL_410540	x
Pango-EPI_ISL_410539-GX-P1E-x-Manisjavanica-2017-GuangxiChina	EPI_ISL_410539	x
Bat-EPI_ISL_1699445-x-RmYN05-Rhmalayanus-2020-YunnanChina	EPI_ISL_1699445	x
Bat-EPI_ISL_1699448-x-RmYN08-Rhmalayanus-2020-YunnanChina	EPI_ISL_1699448	x
Bat-EPI_ISL_1699444-x-RsYN04-Rhstheno-2020-YunnanChina	EPI_ISL_1699444	x
Bat-LC663959.1-x-Rc-mk2-2020-ChibaJapan	LC663959.1	x
Bat-LC663958.1-bRcAce2-Rc-os20-2020-NiigataJapan	LC663958.1	x
Bat-LC556375-bRcAce2-Rc-o319-Rhcornutus-2013-IwateJapan	LC556375	x
Bat-LC663793.1-bRcAce2-Rc-kw8-2020-ShizuokaJapan	LC663793.1	x

Figure S1. 205 sarbecovirus sequences used for bioinformatical analysis. Continued.

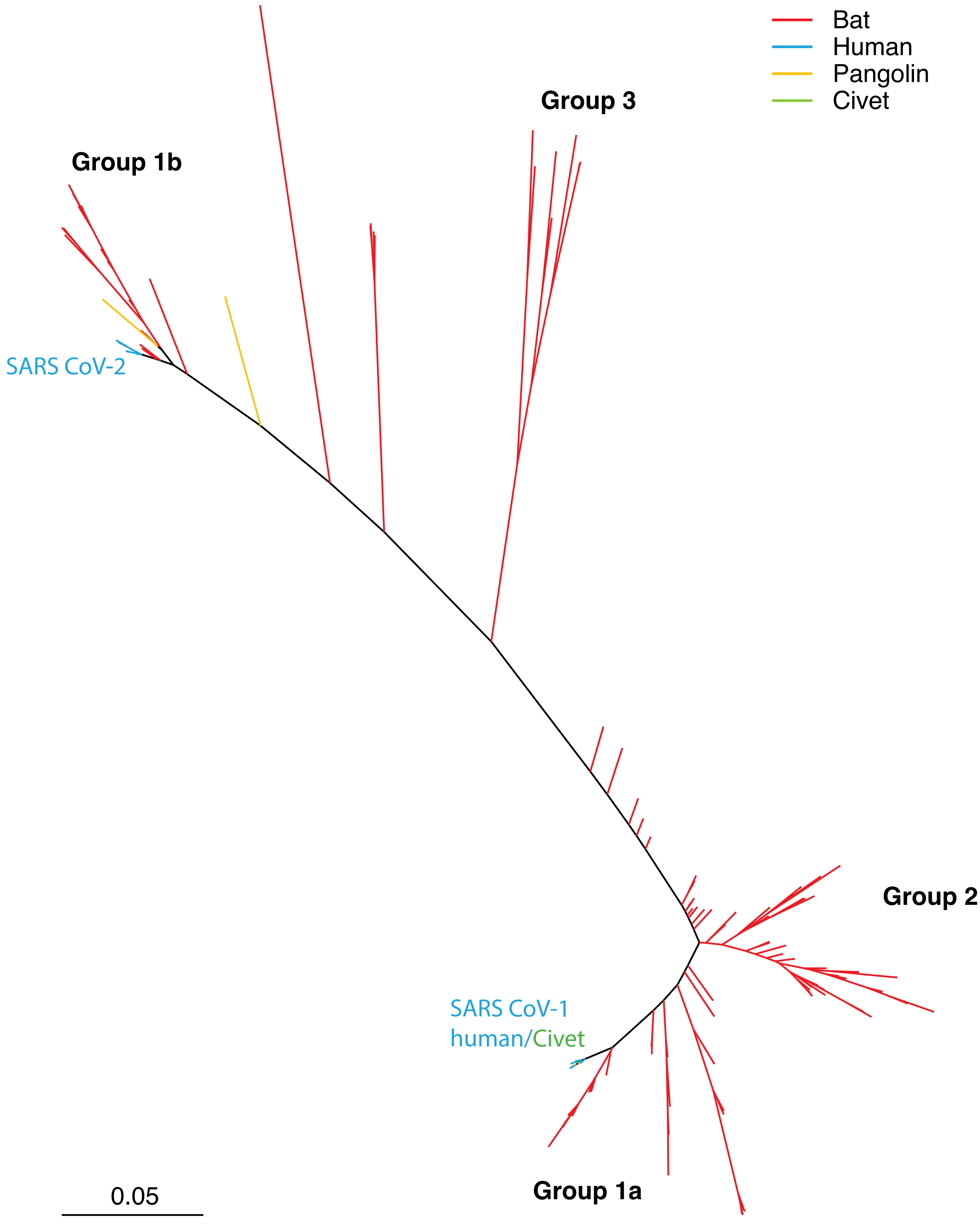

Figure S2. Maximum likelihood phylogenetic tree based on an alignment of the full proteome. It illustrates the relationship of 205 sarbecoviruses from GenBank and GISIAD that span much of the known diversity of these viruses. A sarbecovirus full proteome alignment was first generated and was subsequently codon aligned, translated and optimized in regions of insertions. Only open reading frames were retained, and only sequences that spanned the full proteome were included. This alignment utilized small sequence sets representative of the diversity among highly sampled SARS-CoV-2 and SARS-CoV-1 sequences to avoid oversampling of these relatively highly conserved clades that entered and expanded within the human population.

MNCLDDRCILHCANFNVLFSTVFPPTSFGPLVRKIFVDGVPFVVSTGYHFRELGVVHNQDVNLHSSRLSFKELLVY
AADPAMHAASGNLLLDKRTTCFSVAALTNNVAFQTVKPGNFNKDFYDFAVSKGFFKEGSSVELKHFFFAQDGNAAI
SDYDYYRYNLPTMCDIRQLLFVVEVVDKYFDCYDGGCINANQVIVNNLDKSAGFPFNKWGKARLYYDSMSYEDQDA
LFAYTKRNVIPTITQMNLKYAISAKNRARTVAGVSICSTMTNRQFHQKLLKSIAATRGATVVIGTSKFYGGWHNML
KTVYSDVENPHLMGWDYPKCDRAMPNMLRIMASLVLARKHTTCCSLSHRFYRLANECAQVLSEMVMCGGSLYVKPG
GTSSGDATTAYANSVFNICQAVTANVNALLSTDGNKIADKYVRNLQHRLYECLYRNRDVDTDFVNEFYAYLRKHFS
MMILS**AA**AVVCFNSTYASQGLVASIKNFKSVLYYQNNVFMSEAKCWTETDLTKGPHEFCSQHTMLVKQGDDYVYLP
YPDPSRILGAGCFVDDIVKTDGTLMIERFVSLAIDAYPLTKHPNQEYADVFHLYLQYIRKLHDELTGHMLDMYSVM
LTNDNTSRYWEPEFYEAMYTPHTVLQAVGACVLCNSQTSLRCGACIRRPFLCCKCCYDHVISTSHKLVLSVNPYVC
NAPGCDVTDVTQLYLGGMSYYCKSHKPPISFPLCANGQVFGLYKNTCVGSDNVTDFNAIATCDWTNAGDYILANTC
TERLKLFAAETLKATEETFKLSYGIATVREVLSDRELHLSWEVGKPRPPLNRNYVFTGYRVTKNSKVQIGEYTFEK
GDYGDAVVYRGTTTYKLNVGDYFVLTSHTVMPLSAPTLVPQEHYVRITGLYPTLNISDEFSSNVANYQKVGMQKYS
TLQGPPGTGKSHFAIGLALYYPSARIVYTACSHAAVDALCEKALKYLPIDKCSRIIPARARVECFDKFKVNSTLEQ
YVFCTVNALPETTADIVVFDEISMATNYDLSVVNARLRAKHYVYIGDPAQLPAPRTLLTKGTLEPEYFNSVCRLMK
TIGPDMFLGTCRRCPAEIVDTVSALVYDNKLGGKPCIKVATVQSKMSDVKCTSVVLLSVLQQLRVESSSKLWAQCV
QLHNDILLAKDTTEAFEKMVSLVLLSMQGAVDINKLCEEMLDNRATLQAIASEFSSLPSYAAFATAQEAYEQAVAN
GDSEVVLKKLKKSLNVAKSEFDRDAAMQRKLEKMADQAMTQMYKQARSEDKRAKVTSAMQTMLFTMLRKLDNDALN
NIINNARDGCVPLNIIPLTTAAKLMVVIPDYNTYKNTCDGTTFTYASALWEIQQVVDADSKIVQLSEISMDNSPNL
AWPLIVTALRANSAVKLQNNELSPVALRQMSCAAGTTQTACTDDNALAYYNTTKGGRFVLALLSDLQDLKWARFPK
SDGTGTIYTELEPPCRFVTDTPKGPKVKYLYFIKGLNNLNRGMVLGSLAATVRLQAGNATEVPANSTVLSFCAFAV
DAAKAYKDYLASGGQPITNCVKMLCTHTGTGQAITVTPEANMDQESFGGASCCLYCRCHIDHPNPKGFCDLKGKYV
QIPTTCANDPVGFTLKNTVCTVCGMWKGYGCSCDQLRIPNPLLGLD
1642 amino acids

Figure S3. Amino acid sequence of the COVconsv12 immunogen. Mutations in the RdRp active site are highlighted.

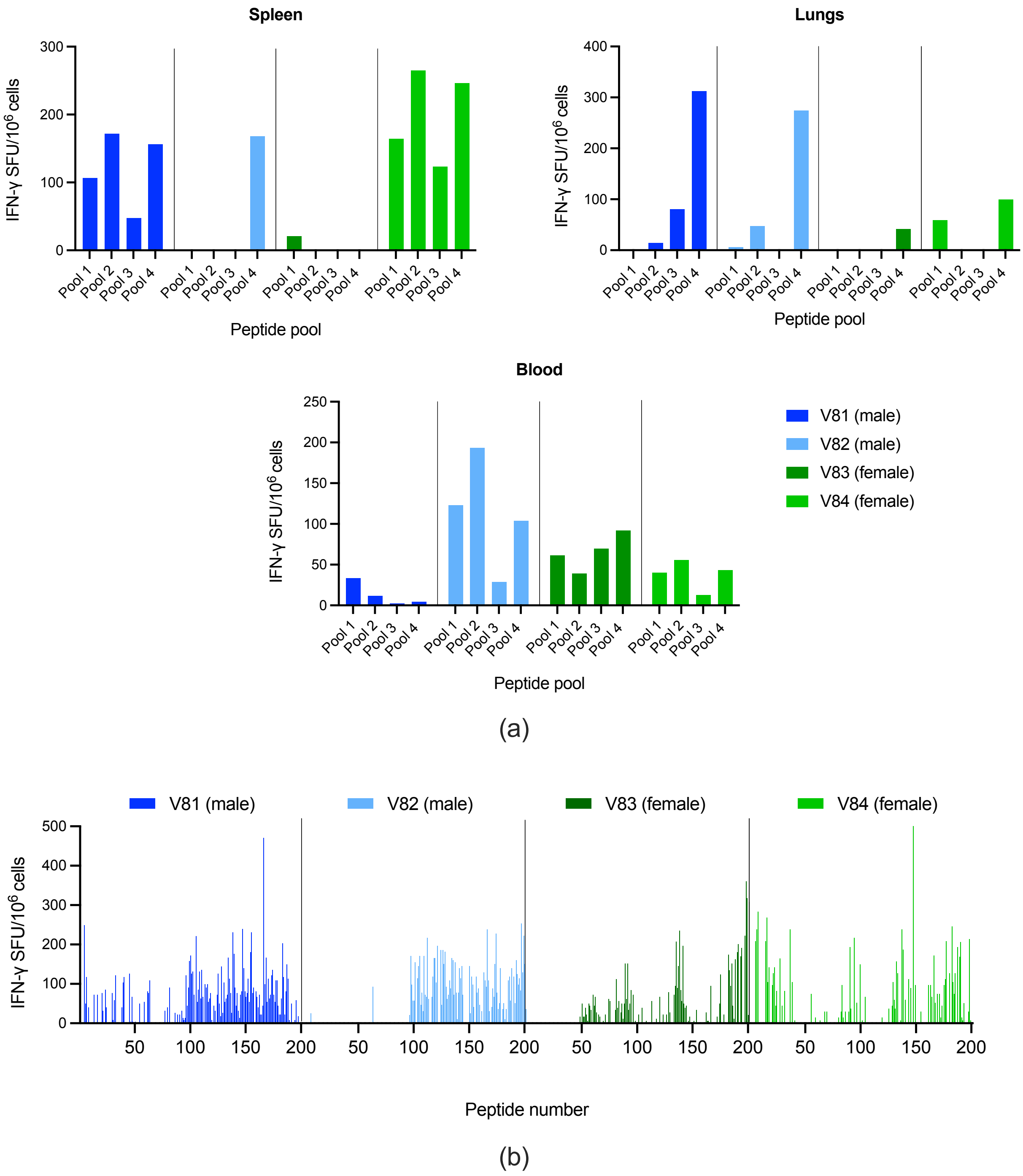

Figure S4. Pilot immunogenicity study of ChAdOx1.COVconsv12 in Syrian hamsters. Four hamsters (2 males & 2 females) were immunised intramuscularly with 2.5 x 10^8 vp of ChAdOx1.COVconsv12 and were sacrificed 13 days later. Spleen, lung and blood cells were isolated and tested in an IFN-γ ELISpot assay (Hamster IFN-γ ELISpot^{BASIC} kit, Mabtech AB, Sweden, Product Code: 3102-2A) as per the vendor's instructions. Cells were restimulated with 201 peptides (18-mers overlapping by 10 amino acids) divided into four pools of ~50 peptides each (**a**). Additionally, splenocytes were restimulated with the 201 individual peptides (**b**).

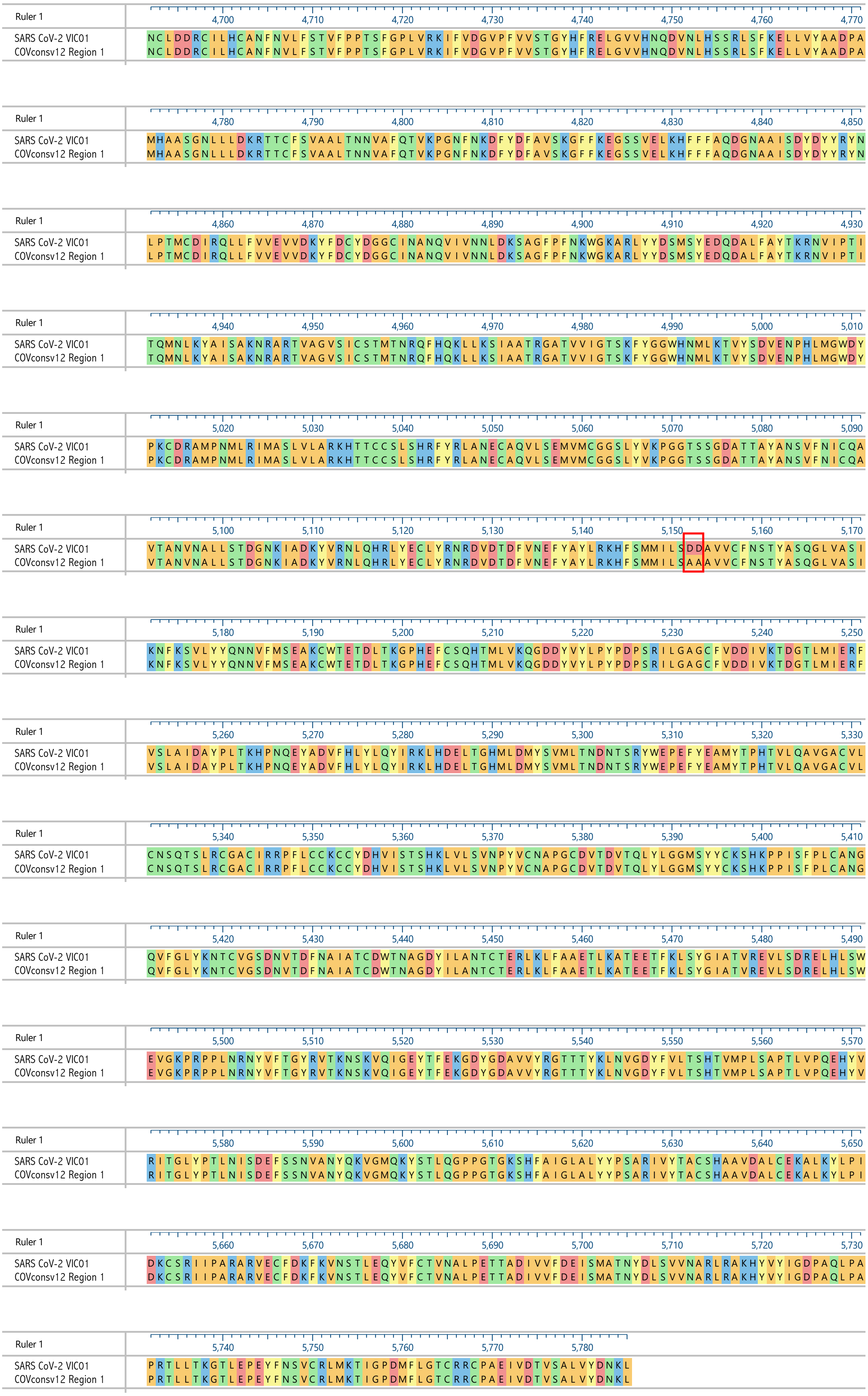

Figure S5. Amino acid alignment of the challenge virus and the vaccine.

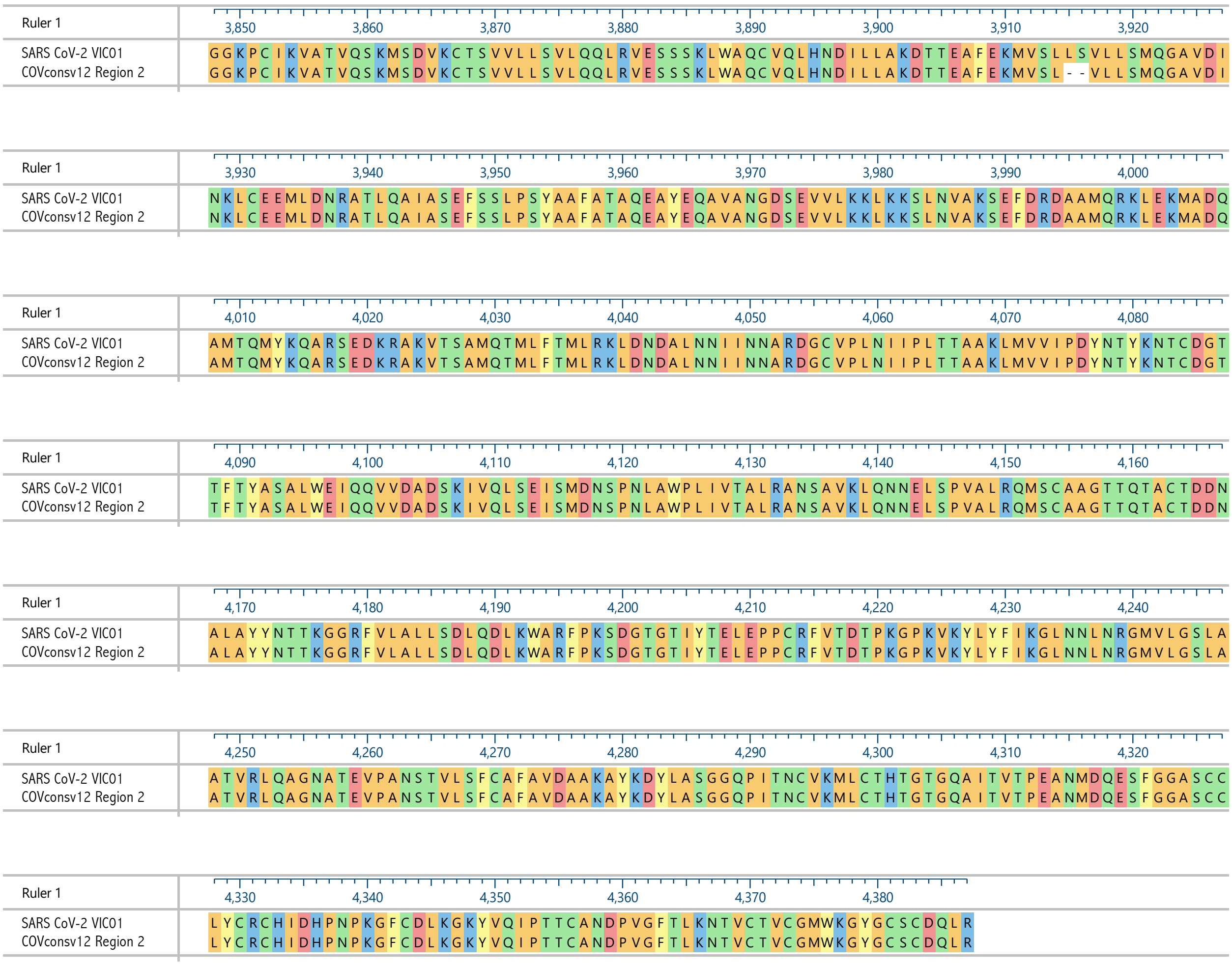

Figure S5. Amino acid alignment of the challenge virus and the vaccine. Continued. SARS-CoV-2 VIC01 (Genbank MT007544) and the COVconsv12 immunogen Region 2 (NS7 - NS10). Amino acid position numbers correspond to those in SARS-CoV-2. The amino acid residues are colour-coded into physico-chemical subgroups: orange - hydrophobic non-polar with alkylgroup; yellow - hydrophobic non-polar with aromatic group; green - hydrophilic polar neutral; red - hydrophilic polar acidic; and blue -hydrophilic polar basic.
